# SANITARY PAD DERMATITIS

**DOI:** 10.4103/0019-5154.57626

**Published:** 2009

**Authors:** Viroj Wiwanitkit

**Affiliations:** *Wiwanitkit House, Bangkhae, Bangkok, Thailand. E-mail: wviroj@yahoo.com*

Sir,

Generally, female menstruation is considered as a normal physiological fertile cycle. Menstruation can be described as the passing of blood via vagina. The passing blood can soak the dress; and sanitary pad has been developed to absorb the passing blood. Modern disposable sanitary pads are becoming available worldwide.[1] Pad usage practices depend on culture, economics, and menstrual physiology.[1] In this article, the author reports an interesting case of sanitary pad dermatitis.

A 38-years-old female patient presented with itching and burning sensation over her perineal area at the contact site with a sanitary pad. The skin lesion presented as red edematous skin with some clear fluid-filled vesicles. No other abnormality was detected through physical examination. The patient noted that she had these symptoms every month on and off and she had even tested herself by using sanitary pads during the non menstruation period and experienced the same symptoms despite using the sanitary pads by various manufactures. This case was diagnosed to be sanitary pad dermatitis.

This case presents an interesting situation. There are three aspects of the above situation: Gynecological, dermatological, and family medicine. As far as the gynecological aspect is concerned, skin lesions at perineum has several implications ranging from benign to malignant diseases. In some cases, biopsy for laboratory investigation is needed.

However, in this case, the skin lesion is strongly related to the use of sanitary pads. It presented on and off during the menstrual cycle of the patient, concordant with the nature of sanitary pad dermatitis.[2,3] A pattern of skin lesion on the contact surface rules out menstruation related skin lesion.[2,3]

As far as the dermatological aspect, is concerned, the pattern is a clear contact dermatitis. There is no need for any provocative test in this case because the patient performed self provocation. Concerning the inductive agent, the material used for the manufacture of sanitary pads is expected to be the main allergen, as there was no change observed when sanitary pad of another manufacturer was used. Indeed, methyldibromo glutaronitrile in sanitary pads is the main inductive agent.[2] Considering the family medicine aspect, the social context in this case is well demonstrated. The Thai and people in many developing countries usually think that talking about menstruation is taboo[4] and this can lead to under-diagnosis of many pathological disorders. In this case, the patient did not allow photography due to this rooted belief. Health education on this topic is recommended. Till now, there is no report on the exact incidence of sanitary pad dermatitis in Thai literature.
